# Larval diet affects mosquito development and permissiveness to *Plasmodium* infection

**DOI:** 10.1038/srep38230

**Published:** 2016-12-02

**Authors:** Inbar Linenberg, George K. Christophides, Mathilde Gendrin

**Affiliations:** 1 Department of Life Sciences, Imperial College London, London SW7 2AZ, UK

## Abstract

The larval stages of malaria vector mosquitoes develop in water pools, feeding mostly on microorganisms and environmental detritus. Richness in the nutrient supply to larvae influences the development and metabolism of larvae and adults. Here, we investigated the effects of larval diet on the development, microbiota content and permissiveness to *Plasmodium* of *Anopheles coluzzii*. We tested three fish diets often used to rear mosquitoes in the laboratory, including two pelleted diets, *Dr. Clarke’s Pool Pellets* and *Nishikoi Fish Pellets*, and one flaked diet, *Tetramin Fish-Flakes.* Larvae grow and develop faster and produce bigger adults when feeding on both types of pellets compared with flakes. This correlates with a higher microbiota load in pellet-fed larvae, in agreement with the known positive effect of the microbiota on mosquito development. Larval diet also significantly influences the prevalence and intensity of *Plasmodium berghei* infection in adults, whereby *Nishikoi Fish Pellets*-fed larvae develop into adults that are highly permissive to parasites and survive longer after infection. This correlates with a lower amount of *Enterobacteriaceae* in the midgut microbiota. Together, our results shed light on the influence of larval feeding on mosquito development, microbiota and vector competence; they also provide useful data for mosquito rearing.

Approximately half of the world population is currently at risk of malaria, caused by infection with protozoan *Plasmodium* parasites^1^. Human malaria is transmitted via bites of infected female *Anopheles* mosquitoes. *Anopheles gambiae* and *A. coluzzii* are the main vectors of *Plasmodium falciparum* malaria in Africa, where 88% of malaria deaths occurred in 2015^1^. When ingested by female mosquitoes in an infectious blood meal, *Plasmodium* parasites undergo sexual reproduction in the midgut lumen, then traverse the peritrophic membrane and the midgut epithelium and differentiate into oocysts upon reaching the basal lamina^2^. In the following one to two weeks, oocysts undergo internal mitotic divisions and maturation until they rupture, releasing thousands of parasites which migrate to the salivary glands and can be transmitted to a human host by mosquito bite.

The oocyst stage is a critical bottleneck of parasite development, which determines if a mosquito will eliminate the parasite or transmit malaria. The success of parasite infection depends on genetic and non-genetic characteristics of each mosquito. First, the mosquito size influences the volume of ingested blood, hence the number of ingested parasites^3^. Moreover, the NF-KB Immune-deficiency (Imd) pathway and the complement-like Thioester-protein 1 (TEP1) pathway clear parasites as they cross the epithelium and as they come into contact with the circulation system, respectively^2^,^4^,^5^,^6^. Finally, the mosquito midgut bacterial community dramatically proliferates within 24 h post blood meal and negatively impacts *Plasmodium* development by inducing immune responses and synthesising reactive oxygen species^6^,^7^,^8^,^9^. The composition of the microbiota may also affect and/or be controlled by *Plasmodium*, as suggested by a positive correlation between the proportion of *Enterobacteriaceae* with a successful *Plasmodium* infection^10^.

Every stage of mosquito development occurs in aquatic conditions, the type of breeding site differing between mosquito species. For instance, *A. gambiae* prefers rain-dependent breeding sites while its twin species *A. coluzzii* mainly breeds in long-lasting water collections linked to human activity, such as rice fields and reservoirs^11^. Larvae feed on organic matter from the environment, notably plant debris, crustaceans, insect scales as well as microorganisms including algae, protozoa and bacteria^12^. The type of soil and food richness in the larval breeding sites are known to influence larval development^13^,^14^. Microbes, notably algae and bacteria, are required for development and are even known to increase development rate^15^,^16^,^17^,^18^. Some bacteria are transmitted to adults and participate in the adult microbiota composition^17^,^19^,^20^,^21^. Nutrient richness at the larval stage also influences the size and metabolism of adult mosquitoes^22^; habitat type also affects the adult permissiveness to malaria parasites^14^, but whether this is mediated by nutrition is as yet unclear.

In a laboratory context, we hypothesized that such effects of larval nutrition and environment may translate in rearing conditions, and more specifically larval diet, affecting the biology of larvae and adult mosquitoes. As diet is known to influence the microbiota composition in metazoans^23^,^24^, we were particularly interested in assessing whether it affects the microbiota of colonised mosquitoes and thus potentially contributes to the high difference in microbiota composition between laboratories^10^,^25^,^26^,^27^. In this study, we analysed the effect of larval diet on the development, the microbiota and the vector competence of *A. coluzzii*. Focusing on three fish diets commonly used in insectaries, we show that these diets differentially influence the speed of larval development, the size of adult mosquitoes and the larval and adult microbiota. Larval diet also significantly impacts prevalence and intensity of infection by *P. berghei*, likely with a microbiota-dependent mechanism.

## Results

### Larval diet influences mosquito development, physiology and permissiveness to *Plasmodium*

**Figure 1:**
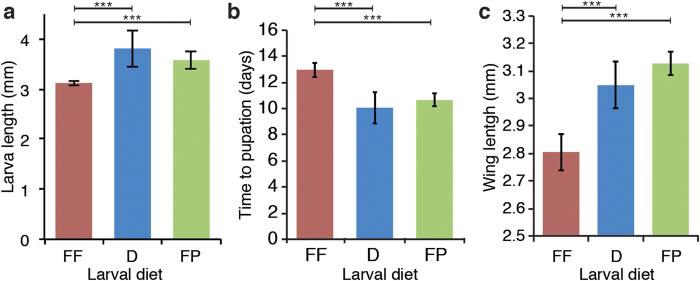
Effect of the larval diet on mosquito development. (**a**) Lengths of larvae on day 7 of feeding on one of the three larval diets: Tetramin fish-flakes (FF), Dr. Clarke’s pool pellets (D) or Nishikoi fish-pellets (FP). (**b**) Average time to pupation of larvae provided with each diet from the first day of feeding. (**c**) Length of adult wings after larval feeding on the three larval diets. Data show averages ±s.e.m. from 3 independent experiments and statistical analyses are ANOVA following lmer model fitting. ***p < 0.001.

As the success and rate of larval development are critically affected by the amount of food present in the water^28^,^29^, we hypothesised that the diet offered to mosquito larvae had an impact on their growth rate and time to pupation. Using Tetramin fish-flakes (‘FF’), commonly used in our laboratory, as a control, we tested two other dietary treatments commonly used for mosquito rearing: Dr. Clarke’s Pool Pellets (‘D’) and Nishikoi fish-pellets (‘FP’). We split 2-day old larvae into three pans, provided them with comparable quantities of food, determined according to the weight of fat and proteins, and monitored their growth by measuring their length 7 days later. Larvae were 22% and 15% longer when reared on the D and FP pelleted foods, respectively, than on the FF flaked diet, indicating that these larvae elongated faster when reared on the D and FP foods (Fig. 1a). Larval development was also faster on D and FP than on FF, as quantified with a 2-to-3-day shorter time to pupation (Fig. 1b). As diet richness during larval development influences the size of the adults, we quantified the wing length as a read-out of the adult size. Larvae fed on both pelleted diets gave rise to significantly bigger adults than larvae fed on flakes (Fig. 1c).

**Figure 2:**
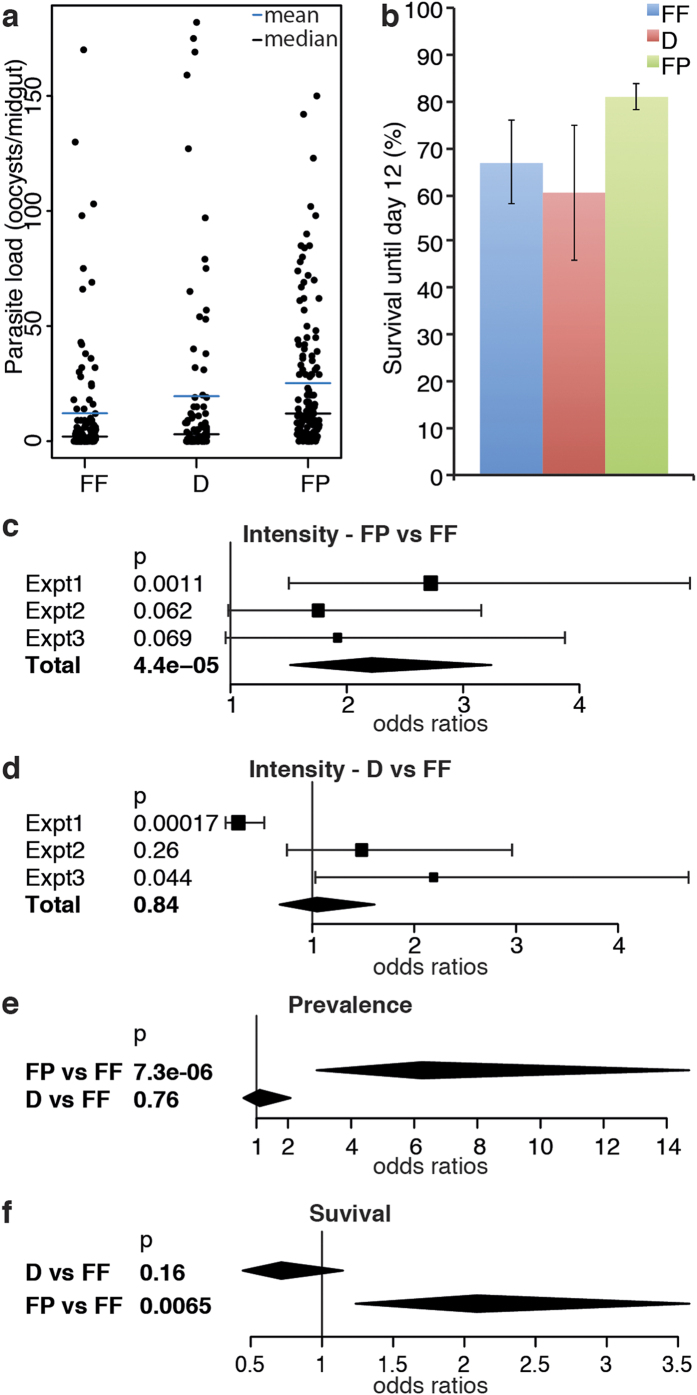
Effect of larval diet on mosquito permissiveness to *P. berghei*. (**a**) Stripchart representation of *P. berghei* infection load in mosquitoes reared as larvae on the three diets. Each dot represents the infection load in an individual mosquito. Pool of 3 independent experiments. (**b**) Proportion of surviving mosquitoes 12 days after *P. berghei* infection, at the time of dissection. Data show average [s.e.m.] of three independent replicates. (**c–f**) Forest plots showing statistical analyses of the experiments displayed in (**a–b**) *i.e.* the effect of the FP (**c**,**e**,**f**) and D (**d–f**) larval diets on *P. berghei* infection intensity (**c,-d**) and prevalence (**e**), and on mosquito survival to day 12 (**f**), using the FF diet as a reference. They were calculated by Wald Z-test on a zero-inflated negative binomial regression (**c,d**) glmmADMB), by ANOVA **X**^2^-test on a logistic regression (**e,f)** glmer). In (**c,d**) squares represent odds ratios of the fixed effect of the diet in an individual experiment (± Confidence Interval, CI). In (**c–f**) diamonds are summaries of all the replicates, their center indicates the fixed effect and their length the CI.

Next, we investigated whether larval diet influences the permissiveness of adults to *Plasmodium*. Females fed as larvae with the three different diets were infected with *P. berghei*, and their infection rate was monitored 12 days later by counting the number of oocysts per gut (intensity) and the proportion of mosquitoes carrying oocysts (prevalence). Females reared on FP were significantly more permissive to *P. berghei* than their siblings reared on FF, as shown by their significantly higher intensity and prevalence of infection (Fig. 2a,c,e; +360% in median intensity, p < 0.001, +33% prevalence, p < 0.001), whilst no significant difference was observed between D and FF (Fig. 2a,d,e; +125% in median intensity, p = 0.84, +6% prevalence, p = 0.76). Interestingly, the proportion of females surviving until the day of dissection was higher in the group reared on FP than in the control (Fig. 2b,f).

Larval nutrition influences larval development and adult physiology through the target-of-rapamycin (TOR) signalling in *Aedes*^22^. TOR signalling is induced after blood feeding and leads to the production of the nutrient transporter *Vitellogenin*; starved larvae produce smaller mosquitoes, which show a reduced *Vitellogenin* expression^22^. As rearing on FP and D diets produced bigger mosquitoes, we examined whether the *Vitellogenin* response to blood feeding was increased in these mosquitoes. We quantified *Vitellogenin* at its expression peak, 24 h after blood feeding, by quantitative PCR^30^. Indeed, *Vitellogenin* transcripts were 2.8-fold more abundant in the midguts of mosquitoes reared on D (D vs FF, p = 0.044), but they were at similar levels in the midguts of FP-reared and FF-reared mosquitoes (Supplementary Fig. S1). This suggests that larval diet indeed affects mosquito metabolism, but in a way that does not directly correlate with permissiveness to *Plasmodium*. As the microbiota is another factor affecting mosquito development^15^,^17^,^18^ and vector competence^31^, we went on to analyse whether the observed effects of larval diet correlated with any change in the microbiota.

### Larval diet impacts the microbiota of larvae and adult mosquitoes

**Figure 3:**
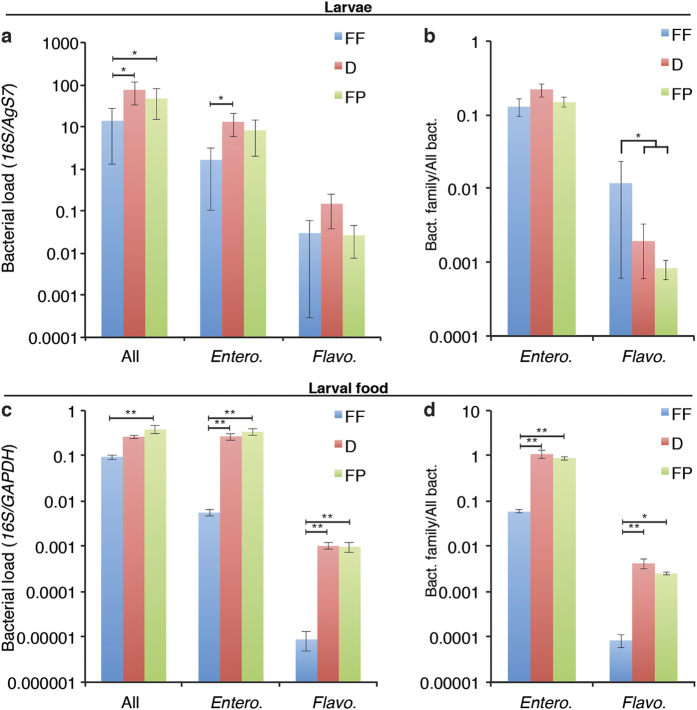
Impact of the larval diet on larval microbiota. (**a**) bacterial load in larvae fed with the three diets, quantified by *16S rRNA* using primers binding to generic sequences conserved in all bacteria (All) or to family-specific sequences (*Entero*. – *Enterobacteriaceae, Flavo.* – *Flavobacteriaceae*) and normalised to the housekeeping mosquito gene *AgS7*. In (**b**) the same family-specific *16S rRNA* quantifications are normalised to the generic *16S rRNA* quantification, and grouped significance bar shows that difference between pelleted and flaked diets. Data show averages (±s.e.m.) from 3 independent replicates and statistical analyses are ANOVA following lmer model fitting, *p <  0.05. (**c**,**d**) Bacterial load in the diet quantified as in (**a**,**b**) respectively. In (**c**) GAPDH from cultured Sf9 cells added prior to RNA extraction was used as a reference gene.

We quantified the microbiota of whole larvae by quantitative PCR on the 16S rRNA bacterial gene seven days after the first feed. The bacterial load in larvae increased by 18-fold when fed with pelleted food (Fig. 3a–d vs FF: 20-fold increase, p = 0.048; FP vs FF: 5.5-fold increase, p = 0.037). We previously showed by 16S sequencing that over 95% of the microbiota of our colony is represented by the *Enterobacteriaceae* and *Flavobacteriaceae* families^25^. Therefore, we monitored the bacterial load of these families by family-specific quantitative PCR as a proxy of the microbiota composition. We observed an increase in *Enterobacteriaceae* when larvae were fed on both pelleted diets (Fig. 3a–d vs FF: 44-fold increase, p = 0.044; FP vs FF: 8.5-fold increase, p = 0.067), whilst the difference in *Flavobacteriaceae* was not significant. When expressing these families as a ratio of the total bacterial load, *Flavobacteriaceae* were present in a lower proportion in pellet-fed larvae than in flake-fed ones (Fig. 3b, 8.5-fold decrease, p = 0.017). As *Asaia sp.* are known to accelerate development when added to the larval breeding water^18^, we also quantified *Acetobacteraceae* but their amount was too close to the detection threshold to allow any reliable quantification. We also quantified bacteria in each diet to investigate whether diets may be the source of larval microbiota. We found more bacteria in the daily amount of food given to larvae in FP than in FF (Fig. 3c, p = 0.0042, Tukey-corrected ANOVA) and marginally significantly more in D than in FF (Fig. 3c, p = 0.066, Tukey-corrected ANOVA). However, the proportions of each bacterial family did not reflect the differences observed in the larval microbiota (Fig. 3d), suggesting that while diet may participate in providing bacteria to larvae, it also influences the microbiota composition by other means.

**Figure 4:**
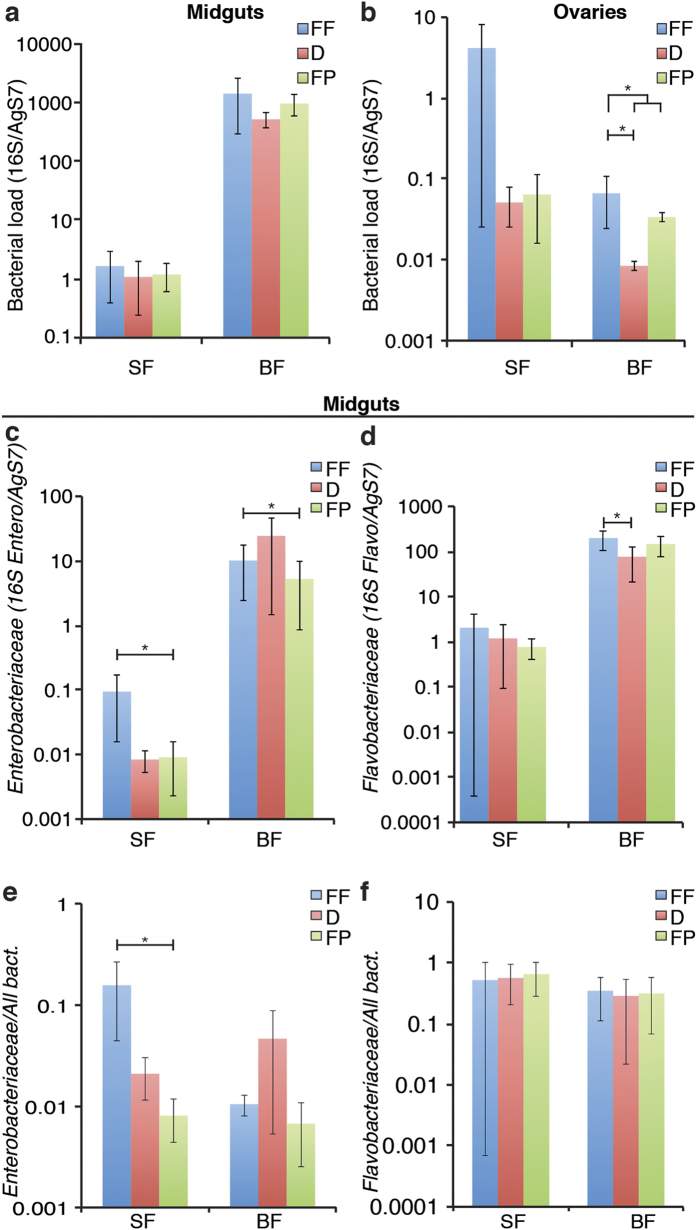
Impact of larval diet on the adult mosquito microbiota. (**a,b**) Bacterial load quantified by qRT-PCR on *16S rRNA* in the midguts (**a**) and ovaries (**b**) of females emerged from larvae fed on the three diets prior to or 24 h after a blood meal (sugar-fed - SF and blood-fed - BF, respectively). (**c–f**) Family-specific bacterial load in adult midguts normalised to *AgS7* (**c,d**) and to the total bacterial load (**e,f**), quantified by qRT-PCR on family specific *16S rRNA* amplicons. SF and BF mosquitoes had significantly different results in (**a)** (***), (**c)** (*), (**d)** (***), (**f)** (**). In (**b**) grouped significance bar shows that difference between pelleted and flaked diets. Data show averages (±s.e.m.) from 3 independent replicates and statistical analyses are ANOVA following lmer model fitting, *p <  0.05, **p < 0.01, ***p < 0.001.

As the adult mosquito microbiota is known to be influenced by the larval breeding site and the larval microbiota^10^,^17^,^21^, we next investigated the impact of larval diet on the midgut microbiota of adult females prior to or after a blood meal (referred to as sugar-fed, SF, and blood-fed, BF, respectively). As shown previously^31^, we observed that the bacterial load in mosquito midguts significantly increased after blood feeding, regardless of the larval diet (Fig. 4a; BF vs SF, 730-fold, p < 0.001). This increase was observed for both bacterial families (Fig. 4c,d; *Enterobacteriaceae* in BF vs SF, 360-fold, p = 0.046; *Flavobacteriaceae* in BF vs SF, 100-fold, p < 0.001), but the relative contribution of *Flavobacteriaceae* to the total bacterial load decreased after the blood meal (Fig. 4f, BF vs SF, 1.8-fold, p = 0.001). Larval diet did not significantly affect the total bacterial load in adult midguts (Fig. 4a, all diets, p = 0.54), but *Enterobacteriaceae* were present at lower levels in the midguts of sugar-fed and blood-fed mosquitoes that received FP as larval diet (Fig. 4c; Sugar-fed FP vs FF, 2.8-fold, p = 0.047; blood-fed FP vs FF, 2.0-fold, p = 0.048). The proportion of *Enterobacteriaceae* within the bacterial microbiota also decreased in sugar-fed midguts of FP-reared mosquitoes (Fig. 4e, FP vs FF, 19-fold, p = 0.022). The amount of *Flavobacteriaceae* decreased in the midguts of blood-fed mosquitoes reared on D (Fig. 4d, D vs FF, 2.6-fold, p = 0.043), but was not affected when reported as a proportion of the total microbiota (Fig. 4f). Again, *Acetobacteraceae* were present at too low levels to allow any comparison.

We also quantified the microbiota in the ovaries, to identify any a vertically-transmissible effect of larval diet on the microbiota. Blood-fed mosquitoes reared on pelleted larval diets appeared to have a lower bacterial load in their ovaries (Fig. 4b, pelleted diets vs FF, 2.9-fold, p = 0.019). In blood-fed mosquitoes, rearing on both pelleted diets also correlated with a lower *Flavobacteriaceae* load and a non-significantly reduced *Enterobacteriaceae* load (Supplementary Fig. S2).

Finally, we looked for statistical interactions between larval diets and tissues or blood-feeding statuses. Considering tissues, we only detected a significant interaction with larval diets on the proportion of *Enterobacteriaceae* in sugar-fed mosquitoes (Table 1, p = 0.04), where the significant difference between FF and FP in the midgut is not observed in ovaries (Figs 4e, S2c). For all other observations, significant effects of diets in one tissue correlate with similar trends in the other tissue, and interaction factors are non-significant (Table 1). Similarly, we found that blood-feeding status only statistically interacts with larval diet when considering the proportion of *Enterobacteriaceae* to the total bacterial load in midguts (Table 1, p = 0.0099). These interactions suggest that the effect of larval diet on the proportion of *Enterobacteriaceae* in sugar-fed midguts is specific, differing to the effect on blood-fed midguts and on ovaries.

## Discussion

In this study, we show that the diet of *Anopheles* larvae influences their rate of growth and development, their microbiota and, after reaching adulthood, their permissiveness to *Plasmodium* infection. Larvae feeding on D or FP were found to grow and develop faster than larvae fed on FF, while they carried a higher bacterial load with a smaller proportion of *Flavobacteriaceae*. Previous studies have shown positive correlations between the presence of a microbiota and the success of larval development^15^,^17^, and between a diet supplemented with *Asaia sp*. and the speed of development^18^. This suggests that the effect of larval diet on larval development that we report here may be mediated by the observed differences in microbiota load and/or composition. Although the exact mechanisms underlying the role of the microbiota on mosquito development are currently unknown, genomic analyses of the bacterial strains and functional studies of other insects suggest that microbiota participate in macromolecule breakdown^32^, synthesise nutrients such as vitamins and essential amino acids^33^ and promote growth and development via the insulin pathway^34^,^35^.

The three diets were matched in protein and fat content in this study, but we cannot rule out that differences in growth and development may be linked with a higher nutrient supply when feeding on D and FP. Indeed, the composition of all three commercial diets was not fully available, in particular energetic values and vitamin contents were missing. Nucleic acids are known to stimulate a feeding response; sterols, which are specific to eukaryotes, are essential for mosquito development^12^. These compounds might be involved in the observed differences in larval growth. As higher food concentration is reported to reduce time to pupation^13^, larvae coming in contact with the pellets may have developed faster due to ingesting a more concentrated food than their siblings fed with flakes, which were more evenly distributed throughout the water surface. In this case, one could expect a higher variance in time to pupation and larval lengths in pellet-fed larvae due to the more uneven accessibility to the pellets, which was not the case.

We also observed a significant effect of larval diet on larva and adult microbiota composition. *Elizabethkingia*, a genus of *Flavobacteriaceae*, is known to dominate the microbiota in colonies of laboratory-reared mosquitoes, including our own^10^,^25^. This is not representative of what is observed in field-collected mosquitoes, where *Elizabethkingia* is indeed prevalent but in low abundance^10^. Our results indicate a lower proportion of *Flavobacteriaceae* in larvae when feeding on both pelleted diets, suggesting that these diets may promote a microbiota that is more relevant to the study of mosquitoes. Although the larval microbiota, and more particularly *Elizabethkingia*, is known to be partially transmitted to adults^17^,^19^,^20^, the proportion of *Flavobacteriaceae* was not affected in adult mosquitoes in this study. More generally, although the larval diets significantly affected the adult mosquito microbiota, the effects observed in adult mosquitoes were not consistent with the effects in larvae. This may be due to differences in the efficiency of transstadial transmission between bacterial strains. For instance, it was previously documented that *Elizabethkingia* is much more efficiently transmitted from pupae to adults than *Pseudomonas*^19^. Some strains of *Enterobacteriaceae* efficiently colonising larvae of D and FP may not be efficiently transmitted to adults, restoring in proportion *Flavobacteriaceae* to the same level as found in adults reared on FF. Rearing mosquitoes on pelleted diets for several generations may lead to more substantial differences in the adult mosquito microbiota.

As previously reported, blood feeding affected the composition of the mosquito microbiota. While both *Enterobacteriaceae* and *Flavobacteriaceae* grew after blood feeding, the proportion of *Flavobacteriaceae* to the total bacterial load decreased. This may be due to kinetics of growth, or to the fact that *Elizabethkingia* is efficiently digested by *A. gambiae*^36^, potentially reducing its growth speed in the midgut. When quantifying bacteria in the midguts and ovaries of mosquitoes, we observed an overall correspondence between the effects on both tissues, where significant differences in microbiota observed in a tissue correlated with a similar trend in the other tissue. This is in agreement with a recent sequencing study of the midgut, ovary and salivary gland microbiota, showing a clustering of the microbiota composition by feeding history rather than by tissue^37^. The effect of diets however differed both between blood-feeding statuses and between tissues in the relative contribution of *Enterobacteriaceae* to the adult microbiota, sugar-fed midguts appearing to be affected differently to blood-fed guts or ovaries. This may reflect an effect of larval diet on *Enterobacteriaceae* strains that are not fast growing after the blood meal and do not cross the gut epithelium.

Our data also indicate that larval diet influences mosquito permissiveness to *Plasmodium* infection. FP-fed larvae produced highly permissive mosquitoes that survived longer after *Plasmodium* infection than the controls fed on FF, whereas feeding on D did not affect adult permissiveness to *Plasmodium* infection or survival. Although an increase in survival might be surprising with an increased infection load, *Plasmodium* infection is known not to inflict much of a fitness cost on mosquitoes in the absence of nutritional stress^38^, as is the case for our mosquitoes fed with fructose *ad libitum*. Thus, our results point to FP mosquitoes being fitter, thus either allowing a higher *Plasmodium* infection or surviving it better. These effects on mosquito infection correlate with a significantly lower amount of *Enterobacteriaceae* in mosquitoes reared on FP. We previously reported a similar combination of a decrease in *Enterobacteriacae*, a higher survival rate and a higher permissiveness to *Plasmodium* when adding a penicillin-streptomycin antibiotic cocktail to the blood meal^25^. As already reported for several strains of *Enterobacteriaceae* including *Enterobacter EspZ* and *Serratia marcescens* HB3^8^,^39^, we can hypothesize that a strain of the *Enterobacteriaceae* present in our mosquito colony may have a direct or indirect anti-parasitic action. These data might appear contrary to the previously reported positive correlation between the relative contribution of *Enterobacteriaceae* in the mosquito microbiota and the success of parasite infection^10^,^40^. However, the effect of bacteria from the microbiota is known to be highly strain-specific.

In conclusion, our results provide new insights into the effect of larval diet on mosquito development, microbiota and permissiveness to malaria parasites. They are also of technical interest in mosquito rearing, showing that faster development can be achieved when using the pelleted diets D and FP, and that FP produces mosquitoes with a higher *Plasmodium* infection rate.

## Methods

### Ethics Statement

Animal use was carried out in accordance with the UK Animals (Scientific Procedures) Act 1986. The protocol for infecting mosquitoes with *P. berghei* by blood feeding on parasite-infected mice was carried out under the UK Home Office License PPL70/7185 awarded in January 2010. The procedures are of mild to moderate severity and the number of animals used are minimised by incorporation of the most economical protocols. Protocols were implemented following approval by the Imperial College Ethical Review Committee.

### Mosquito Colony and Maintenance

Experimental work was performed on the *An. coluzzii* Ngousso colony established in 2006 from field-collected mosquitoes from Cameroon, maintained on human blood at 27 °C and 70% humidity, with 12 h:12 h light/dark cycle. Adult mosquitoes were fed with a 5% fructose solution and larvae were kept in distilled water. Larval generations prior to those obtained for dietary treatments were fed Tetra FF ground to 1–2 mm fragments, the control diet used in this study. Pupae were collected daily, placed in glass beakers with distilled water and transferred to cages.

### Larval feeding

For each dietary treatment, 400 (±10%) 48 h-old unfed *An. coluzzii* first instar larvae were counted manually or using a COPAS larvae sorter (Union Biometrica, USA) and put into a plastic pan. Each larval pan was fed on one of three conventional fish-based diets – Tetra Tetramin fish-flakes (‘FF’), Dr. Clarke’s Pool Pellets (‘D’), and Nishikoi Fish Pellets (‘FP’). Nutritional summaries of these diets are shown in Table 2. As reported in Table 2, diets were provisioned in quantities providing comparable levels of nutritional constituents in each feed. FF were slightly blended to reduce the size of the flakes, as commonly performed in our laboratory for larval rearing.

### Larval length measurements

Twenty 9-day-old larvae (from the time of egg collection) were collected from each treatment of 3 independent experiments, and placed into individual wells of a 96-well plate for imaging at 20x magnification. The whole body length was measured from the top of the head to the tip of the abdomen using Fiji^41^.

### Wing length measurements

Twenty adults were collected from each treatment of 4 independent experiments. One wing of each individual was removed and taped onto a sheet of paper. After scanning the paper, wing lengths were measured using Fiji^41^.

### Microbiota and gene expression analysis

Twenty individuals (9-day-old larvae, 3–5 day-old females prior to or 24 h after blood feeding) were collected from each treatment of 4 independent experiments, suspended in 70% ethanol for 3–5 minutes, then washed 3 times in PBS as performed previously^25^. Adult midguts and ovaries were dissected and sampled in 2 groups of 10 tissues. Forceps and slides were cleaned with distilled water and ethanol between each sample. To quantify bacteria in food, a daily (d7+) amount of food was homogenized in 5 mL sterile water, then 1 mL was sampled, spun at 800 g for 5 min and the supernatant was discarded. 4 replicates were monitored for each diet. In order to normalise the amount of RNA, a suspension of Sf9 cells from a confluent culture of a 175 cm^2^ flask was split into all 12 samples.

RNA was extracted from whole larvae, adult tissues and pelleted water contents with Trizol and chloroform, precipitated using isopropanol and washed twice in 70% ethanol. Reverse transcription (Takara) was performed using PrimeScript RT Enzyme Mix I with 650 ng RNA following the manufacturer’s instructions. The bacterial *16S* rRNA gene was used for quantification by qRT-PCR performed on a LightCycler 480 (Roche), using SYBR *Premix Ex Taq* kit (Takara) following the manufacturer’s instructions. The bacterial load and composition was assessed using *16S* rRNA gene primers general to all bacteria and specific to bacterial families known to colonise mosquitoes in the insectary insectary, respectively (Table S1 and ref. 25). Bacterial load and *Vitellogenin* expression are presented as ratios of the target gene to the reference *Anopheles* house-keeping gene *40S ribosomal protein S7* (S7), while bacterial load in food was quantified as a ratio of the *GAPDH* (glyceraldehyde-3-phosphate-dehydrogenase) house-keeping gene in Sf9 cells.

### *Plasmodium* infections

An 8–12-week-old CD1 female mouse was intraperitoneally injected with 100 μl 5O7 *P. berghei*-infected blood and kept for two days to allow parasite growth and gametocyte differentiation. After checking that parasitemia was between 3 and 8%, the mouse was intramuscularly injected with 4 μL. g^−1^ of an anesthetic solution (Rompun 0.33%, ketamine 33 mg. mL^−1^ in PBS) and used to blood feed three cups of mosquitoes in turns of 5–10 min. Mosquitoes were kept for 12 days at 20 °C to allow parasite development until the oocyst stage. Then, dead mosquitoes were removed and scored and all surviving mosquitoes were dissected for oocyst counts using a fluorescent microscope. For each experiment, at least 50 mosquitoes per condition were offered a blood meal and non-engorged mosquitoes were removed 24 h later. On average, the oocysts of 36 (±3.6 s.e.m.) mosquitoes per condition per experiment were counted. Differences in median intensities and prevalence are mentioned in the text as averages of the effect from each replicate; that is, (data(test diet) - data(control diet))/data(control diet).

### Statistics

Statistical analyses were performed by generalised linear mixed models (GLMM) in R (version 3.2.3). An ANOVA **X**^2^-test on a logistic regression (glmer) was performed on factorial data (prevalence, survival). A Wald Z-test on a zero-inflated negative binomial regression (glmmADMB) was performed for oocyst counts. An ANOVA **X**^2^-test on a common linear regression (lmer) was performed on qPCR data (*16S*, gene expression), larva length, time to pupation and wing length. GLMM analyses decompose data into a fixed component (here larval diets) and a random component (here replicate experiments). Odds ratios presented in Fig. 2 are the exponential of the regression coefficients. Sample size was chosen according to the standard protocols (oocyst counts, gene expression, microbiota analyses) or estimated according to mosquito rearing experience (larval development). Mosquitoes were excluded if they did not blood feed, if they burst during dissection (microbiota analyses, gene expression) or if they died (as larvae or adults) during the experiment.

## Additional Information

**Accession Codes:** The Vectorbase (www.vectorbase.org) accession numbers of the genes mentioned in this study are: AgS7 – AGAP010592; *Vitellogenin* – AGAP004203.

**How to cite this article**: Linenberg, I. *et al*. Larval diet affects mosquito development and permissiveness to *Plasmodium* infection. *Sci. Rep.* **6**, 38230; doi: 10.1038/srep38230 (2016).

**Publisher’s note:** Springer Nature remains neutral with regard to jurisdictional claims in published maps and institutional affiliations.

## Supplementary Material

Supplementary Figures and Table 1

## Figures and Tables

**Table 1:** Statistical analysis of the interactions between larval diet and tissue or blood feeding status.

Samples	Interaction	*16S/AgS7*	*Flavo/AgS7*	*Entero/AgS7*	*Flavo/16S*	*Entero/16S*
All adult samples	tissue x diet	0.55	0.57	0.36	0.28	0.72
	BF status x diet	0.48	0.52	0.50	0.24	0.74
Midguts	BF status x diet	0.25	0.30	0.47	0.81	**0.0099**
Ovaries	BF status x diet	0.22	0.30	0.86	0.17	0.39
SF	tissue x diet	0.45	0.75	0.18	0.47	**0.041**
BF	tissue x diet	0.27	0.30	0.38	0.10	0.33

The p-values of ANOVA following lmer model fitting are indicated and significant p-values are shown in bold.

**Table 2:** Summary of nutritional constituents of larval diets and of feeding regimes.

	Fish flakes - FF	Dr Clarke’s pellets - D	Nishikoi Fish pellets - FP
**Nutritional Constituent (% w/w; mg/ feed, days 7+)**			
Protein	47.0; 23.5	34.0; 23.4	26.0; 26.4
Oils/Fats	10.0; 5.0	5.5; 3.8	4.0; 4.1
Fibre	3.0; 1.5	3.0; 2.1	unknown
Ash	unknown	7.0; 4.8	8.2; 8.3
Unknown	40; 20 mg/feed	50.5; 34.8 mg/feed	61.8; 63.0 mg/feed
**Feeding regime**			
Days 1–6	25 mg/day	1 pellet (69 mg)/2 days	1 pellet (102 mg)/2 days
Days 7+	50 mg/day	1 pellet/day	1 pellet/day

Ash consists of inorganic matter. Foods contained vitamins and provitamins although these were of unknown quantity and not consistently specified. Feeding regime was designed to provide comparable levels of proteins and fats between diets in each feed. Larvae were not fed throughout weekends and instead were given a 2-day worth feed on Fridays.
