# Supplementary material for: Larval diet affects mosquito development and permissiveness to Plasmodium infection
Source: Sci Rep. 2016 Dec 2;6:38230. doi: 10.1038/srep38230 (PMC5133635; doi:10.1038/srep38230)
Supplement: Supplementary Figures and Table 1 [file srep38230-s1.pdf]

Larval diet affects mosquito development and permissiveness to *Plasmodium* infection – Supplementary information

Inbar Linenberg, George K. Christophides and Mathilde Gendrin

Supplementary figures

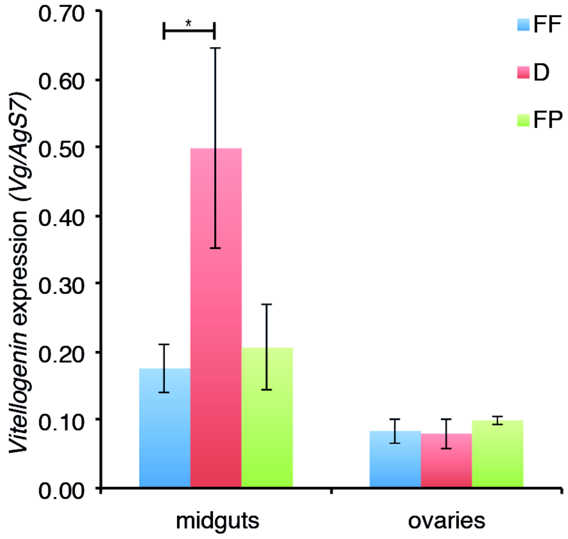

Supplementary figure S1. Impact of larval diet on *Vitellogenin* expression after blood meal. qRT-PCR analysis of *Vitellogenin* expression in the midguts and ovaries of female mosquitoes 24 h after a bloodmeal. Transcript quantifications are normalised to *AgS7*. Data show averages (±s.e.m.) from 3 independent replicates and statistical analyses are ANOVA following lmer model fitting, * - p< 0.05.

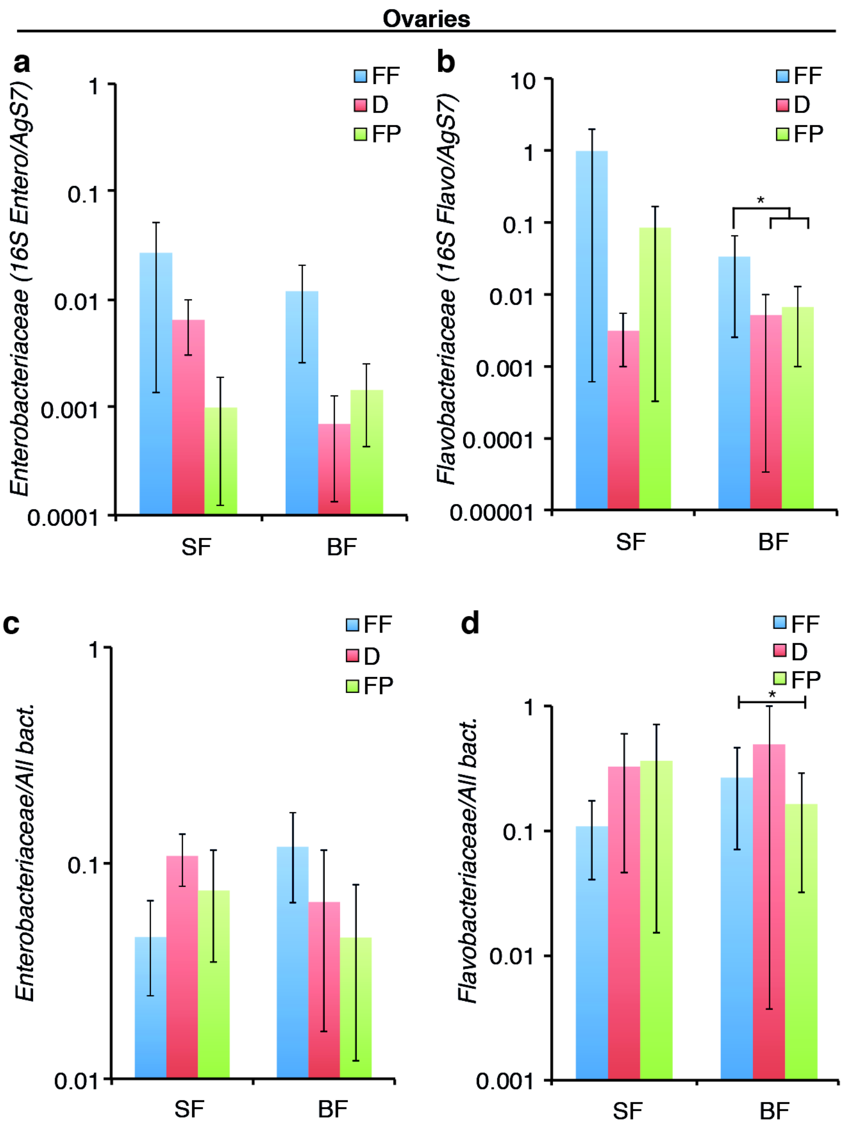

Supplementary figure S2. Impact of larval diet on the composition of the ovary microbiota. Family-specific bacterial load in adult ovaries normalised to *AgS7* (**a-b**) and to the total bacterial load (**c-d**), quantified by qRT-PCR on family specific *16S rRNA* amplicons. Data show averages (±s.e.m.) from 3 independent replicates and statistical analyses are ANOVA following lmer model fitting, * - $p < 0.05$.

Supplementary table

Gene	Sequence F	Sequence R
S7	GTGCGCGAGTTGGAGAAGA	ATCGGTTTGGGCAGAATGC
16S	TCCTACGGGAGGCAGCAGT	GGACTACCAGGGTATCTAATCCTGTT
16S, *Enterobacteriaceae*	CGTGCTACAATGGCATATACAAAGAGAAG	AGCATTCTGATCTACGATTACTAGCGATTC
16S, *Flavobacteriaceae*	TAAGGTTGAAGTGGCTGGAATAA	GTCCATCAGCGTCAGTTAAGACT
16S, *Acetobacteraceae*	GTGCCGATCTCTAAAAGCCGTCTCA	TTCGCTCACCGGCTTCGGGT
Vitellogenin	CTGGTCTATCCGTTCGCCTC	GACGTCGCTGTTGCGAATAC
GAPDH - Spodoptera frugiperda	CGGTGTCTTCACAACCACAG	GAGAGGAGCGAGACAGTTGG

Table S1 – Primer sequences used in qRT-PCR analysis of bacterial microbiota.
